# Effects of Zingiberaceae-derived interventions on memory-related and other cognitive outcomes in adults: a systematic review and meta-analysis

**DOI:** 10.3389/fnut.2026.1834167

**Published:** 2026-05-11

**Authors:** Desirée Victoria-Montesinos, Pilar Zafrilla, Pura Ballester, Ana María García-Muñoz

**Affiliations:** Faculty of Pharmacy and Nutrition, Universidad Católica De Murcia (UCAM), Murcia, Spain

**Keywords:** cognition, cognitive aging, curcumin, ginger, meta-analysis, Zingiberaceae

## Abstract

**Introduction:**

Cognitive impairment and age-related cognitive decline are major public health concerns, and nutraceutical strategies targeting modifiable biological pathways have attracted growing interest. Compounds derived from the Zingiberaceae family, including curcumin, turmeric, ginger, and related preparations, have been investigated for their potential neuroprotective effects, but their clinical impact on specific cognitive domains remains unclear. This systematic review and meta-analysis aimed to evaluate the effects of Zingiberaceae-derived interventions on memory-related and other cognitive outcomes in adults.

**Methods:**

A systematic search was conducted in MEDLINE, Cochrane Central Register of Controlled Trials, Web of Science, and Scopus from inception to March 2026. Randomized controlled trials evaluating orally administered Zingiberaceae-derived interventions in adults and reporting validated cognitive outcomes were included. Cognitive outcomes were grouped into memory-related outcomes, executive function and processing speed, global cognition, and attention or inhibitory control, with memory-related outcomes prespecified as the primary outcome.

**Results:**

Eighteen randomized, double-blind, placebo-controlled clinical trials were included in the qualitative synthesis, and domain-specific meta-analyses were performed when at least three studies were available. In the pooled analysis, Zingiberaceae-derived interventions showed a statistically significant improvement in memory-related outcomes (standardized mean difference = 0.57; 95% confidence interval: 0.13 to 1.02), whereas no significant pooled effects were observed for executive function and processing speed, global cognition, or attention or inhibitory control. Sensitivity analyses were consistent with the main findings.

**Discussion:**

Overall, Zingiberaceae-derived interventions may improve memory-related outcomes, but the evidence is very uncertain due to substantial heterogeneity, the small number of studies, risk of bias in some trials, and variability in populations, formulations, and outcome measures. These findings indicate that their cognitive effects may be domain-specific and context-dependent, highlighting the need for larger, well-designed trials using standardized cognitive endpoints and bioavailable formulations.

**Systematic review registration:**

https://www.crd.york.ac.uk/PROSPERO/view/CRD420261325966, identifier CRD420261325966.

## 1 Introduction

Cognitive impairment, encompassing deficits in memory, attention, executive function, and processing speed, represents one of the most pressing global public health challenges associated with population aging. The prevalence of dementia is projected to increase substantially over the coming decades, with current estimates indicating that more than 55 million people worldwide live with dementia, and nearly 10 million new cases occur annually (1). Complementing these estimates, data from the Global Burden of Disease (GBD) 2021 study indicate that the prevalence of Alzheimer’s disease (AD) and related dementias (ADRD) among adults aged ≥65 years increased by approximately 160% between 1991 and 2021, rising from 18.7 million to 49 million affected individuals globally (2). During the same period, mortality more than doubled, and in 2021 ADRD accounted for an estimated 8 million deaths and over 25 million disability-adjusted life years (DALYs). Notably, women represent a disproportionately affected group, accounting for nearly two-thirds of prevalent cases (2).

Beyond diagnosed dementia, age-related cognitive decline and subjective cognitive complaints are highly prevalent in community-dwelling older adults and are associated with reduced quality of life, increased dependency, institutionalization, and substantial socioeconomic burden. Cognitive impairment should be understood along a clinical continuum that ranges from normal cognitive functioning and subjective cognitive complaints to mild cognitive impairment (MCI) and dementia. This clinical severity continuum is distinct from the underlying etiology or pathology, which may include neurodegenerative, cerebrovascular, metabolic, nutritional, psychiatric, inflammatory, or mixed causes. MCI is therefore clinically relevant because it identifies an intermediate level of cognitive impairment, but it should not be equated with a specific etiology. Although some individuals with MCI progress to dementia due to AD or other underlying etiologies, others remain stable, improve, or have cognitive impairment driven by non-AD mechanisms (3).

From a pathophysiological perspective, cognitive decline is multifactorial and involves neuroinflammation, oxidative stress, mitochondrial dysfunction, cerebrovascular impairment, amyloid-β accumulation, tau hyperphosphorylation, and synaptic dysregulation (4). Importantly, these mechanisms are not mutually exclusive, and mixed-etiology pathology is common, particularly in older adults. This biological heterogeneity has driven growing interest in preventive and adjunctive strategies targeting modifiable pathways that may contribute to cognitive dysfunction across different clinical contexts.

In parallel, there has been increasing scientific interest in nutraceutical and dietary interventions aimed at modulating mechanisms implicated in cognitive aging. Several systematic reviews and meta-analyses have evaluated the effects of dietary polyphenols on cognitive outcomes, suggesting modest but domain-specific benefits, particularly in memory and executive function, although with considerable heterogeneity (5, 6). Importantly, polyphenol bioavailability, interindividual variability, and baseline cognitive status appear to influence clinical outcomes.

Within this context, compounds derived from the Zingiberaceae family have attracted attention due to their anti-inflammatory, antioxidant, and neuroprotective properties. Preclinical evidence suggests that curcumin modulates nuclear factor kappa B (NF-κB; Nuclear Factor kappa-light-chain-enhancer of activated B cells), reduces oxidative stress markers, interferes with amyloid aggregation, and influences synaptic plasticity pathways (7, 8). Similarly, ginger-derived compounds have demonstrated anti-inflammatory and antioxidative effects, along with potential neuroprotective activity in experimental models (9).

However, curcumin presents well-recognized pharmacokinetic limitations, including poor oral bioavailability, rapid metabolism, and low systemic concentrations following ingestion (10). This has led to the development of enhanced formulations (e.g., lipidated, phytosomal, nanoparticle-based, or fiber-complexed preparations) designed to improve absorption and tissue distribution (11). The clinical relevance of these formulations has been explored in randomised controlled trials (RCTs), including studies in healthy older adults reporting improvements in working memory and attention following lipidated curcumin supplementation (12, 13).

Nevertheless, human evidence remains inconsistent. A recent systematic review and meta-analysis evaluating curcumin supplementation and cognitive outcomes reported modest, domain-specific improvements, while failing to demonstrate robust effects on global cognition (14). Substantial heterogeneity was observed across trials, likely driven by differences in participant characteristics, intervention duration, formulation bioavailability, and the cognitive instruments employed. Additionally, a higher incidence of adverse events, predominantly mild gastrointestinal symptoms, was reported in curcumin groups compared with placebo (14). Earlier systematic reviews have similarly highlighted small sample sizes, short intervention periods, inconsistent endpoint selection, and limited reporting of formulation characteristics as key methodological limitations (15, 16).

In contrast, clinical research on ginger-derived compounds remains comparatively sparse. Although individual randomized trials have reported improvements in memory and attention domains in healthy middle-aged adults and in individuals with mild cognitive impairment (17), these findings have not been comprehensively synthesized within a broader phytochemical framework. To date, most systematic reviews have focused exclusively on curcumin, leaving other Zingiberaceae-derived bioactives underrepresented in evidence syntheses.

Importantly, several gaps persist in the current literature. First, prior reviews have often prioritized global cognition as a composite endpoint, without specifically examining memory-related outcomes as a primary outcome, despite its central relevance in early cognitive decline and prodromal AD. Second, formulation-related differences are rarely examined systematically. Third, populations with distinct baseline cognitive statuses (healthy aging, subjective cognitive complaints, mild cognitive impairment, metabolic comorbidities) are frequently pooled, limiting mechanistic and clinical interpretation. Finally, safety and tolerability data are inconsistently integrated into efficacy-focused syntheses.

Given these limitations, a structured evaluation of controlled clinical trials specifically addressing memory-related and cognitive outcomes across Zingiberaceae-derived interventions is warranted. Although cognitive aging and early neurodegenerative stages provide a major clinical context for this field, cognitive symptoms and domain-specific impairments are also relevant across a broader range of adult clinical and non-clinical settings. The present systematic review therefore aims to comprehensively assess the effects of curcumin, turmeric, ginger, and related Zingiberaceae-derived preparations on memory performance and broader cognitive function in adults, with memory-related outcomes defined *a priori* as the primary outcome. By explicitly considering formulation characteristics, population heterogeneity, and safety reporting, this review seeks to clarify the consistency, magnitude, and clinical relevance of current evidence and to identify priorities for future research.

## 2 Materials and methods

### 2.1 Study design and protocol registration

This systematic review was conducted in accordance with the Preferred Reporting Items for Systematic Reviews and Meta-Analyses (PRISMA 2020) statement (18). The methodological framework was predefined and prospectively registered in the International Prospective Register of Systematic Reviews (PROSPERO; registration number CRD420261325966). The protocol specified the research question, eligibility criteria, outcome hierarchy, and planned analytical strategy prior to study identification and selection, thereby reducing the risk of *post hoc* methodological decisions and enhancing transparency and reproducibility.

### 2.2 Eligibility criteria

Eligibility criteria were established *a priori* using the Population, Intervention, Comparator, Outcomes, and Study design (PICOS) framework. Studies were eligible if they included adult participants aged 18 years or older. Both healthy individuals and clinical populations were considered, provided that cognitive outcomes were assessed using validated neuropsychological instruments. Eligible populations therefore encompassed adults across different baseline cognitive status categories, including cognitively healthy middle-aged and older adults, individuals with subjective cognitive complaints, individuals with MCI, and individuals with dementia. Studies were also eligible when participants had a defined clinical condition or potential etiological contributor to cognitive dysfunction, such as AD, metabolic or chronic disorders, mood disorders, chemotherapy-related cognitive impairment, chronic kidney disease, or other adult clinical contexts, provided that cognitive performance was explicitly assessed using validated neuropsychological instruments.

Interventions of interest consisted of orally administered preparations derived from the Zingiberaceae family, including curcumin, turmeric (*Curcuma longa*) extracts, ginger (*Zingiber officinale*) extracts, and standardized formulations enriched in bioactive compounds such as 6-gingerol or 6-shogaol. Enhanced-bioavailability formulations were considered eligible. Studies investigating multi-component supplements were included only when the Zingiberaceae-derived compound represented the principal active ingredient and its independent contribution to cognitive outcomes could be reasonably interpreted. Comparators included placebo, active control interventions, standard care, or non-supplemented control groups.

Cognitive outcomes were prespecified and classified according to neuropsychological domains of relevance in cognitive aging and early neurodegeneration. The review focused on memory-related outcomes, executive function and processing speed, global cognition, and attention or inhibitory control. Memory-related outcomes, encompassing working memory, verbal learning, immediate and delayed recall, recognition, visual memory, and other related memory measures, was defined *a priori* as the primary outcome due to the clinical relevance of memory function in early cognitive decline and prodromal AD. Executive function and processing speed included tasks assessing cognitive flexibility, set-shifting, planning, psychomotor speed, and timed performance measures. Global cognition referred to composite or screening instruments such as the Mini-Mental State Examination (MMSE) or Montreal Cognitive Assessment (MoCA). Attention and inhibitory control included sustained attention and response inhibition paradigms. When multiple measures within a given domain were reported, the prespecified primary endpoint of the study was prioritized; if not clearly indicated, the most clinically validated and widely used instrument was selected. Outcomes outside these prespecified domains were described narratively but were not considered central endpoints. Safety and tolerability data, including adverse events and withdrawals related to side effects, were also extracted and synthesized descriptively.

Randomized controlled trials, including parallel and crossover designs, were eligible for inclusion. Observational studies, non-randomized controlled studies, case reports, animal experiments, and *in vitro* investigations were excluded.

### 2.3 Information sources and search strategy

A comprehensive search strategy was developed in accordance with the registered protocol. Electronic database searches were conducted in MEDLINE (via PubMed), the Cochrane Central Register of Controlled Trials (CENTRAL), Web of Science (WoS) and Scopus. The final electronic database search was completed on 7 March 2026. No restrictions were applied regarding publication date or language in order to maximize retrieval sensitivity. The search strategy combined controlled vocabulary terms and free-text keywords related to Zingiberaceae-derived compounds, including “curcumin,” “turmeric,” and “ginger,” with terms related to cognitive function, such as “memory,” “cognition,” “cognitive function,” “executive function,” and related synonyms. The complete search strategy for all databases is presented in Supplementary Table 1. Reference lists of eligible articles and relevant systematic reviews were manually screened to identify additional potentially relevant trials. Only peer-reviewed published studies were considered for inclusion.

### 2.4 Study selection

All retrieved records were imported into reference management software, and duplicate entries were removed prior to screening. Titles and abstracts were independently screened by two reviewers (DV-M and AMG-M) against the predefined eligibility criteria. Studies considered potentially relevant proceeded to full-text evaluation. Full-text screening was conducted independently by the same reviewers, and any disagreements were resolved through discussion and consensus. When necessary, a third reviewer (PB) was consulted to reach agreement. Reasons for exclusion at the full-text stage were documented to ensure transparency of the selection process.

### 2.5 Data extraction

Data extraction was performed independently by two reviewers (AMG-M and DV-M) using a standardized extraction form developed prior to data collection. Extracted variables included study characteristics such as first author, year of publication, country, study design, and duration; participant characteristics including sample size, mean age, sex distribution, and baseline cognitive status; and detailed intervention characteristics, including botanical source, preparation type, commercial or proprietary formulation when reported, standardization to specific bioactive compounds, daily dose, dosing schedule, intervention duration, and timing of cognitive assessment or follow-up. Intervention preparations were classified, when possible, as conventional powder or food-based preparations, standard extract capsules, enhanced-bioavailability curcumin formulations, ginger-derived preparations, or other Zingiberaceae-derived commercial preparations. Characteristics of the comparator and cognitive outcomes according to the prespecified domains were also extracted. When available, baseline and post-intervention means and standard deviations were recorded. Safety data, including adverse events and discontinuations, were also extracted. When information required for data extraction or quantitative synthesis was unavailable, unclear, or could not be derived from the published report, attempts were made to contact corresponding authors for clarification. When aggregate data were available but the published report indicated substantial attrition, exclusion of randomized participants, or incomplete endpoint contribution, this information was considered in the risk-of-bias assessment.

### 2.6 Risk of bias assessment

Risk of bias for randomized controlled trials was assessed using the Cochrane Risk of Bias 2 (RoB 2) tool (19, 20). This tool evaluates bias arising from the randomization process, deviations from intended interventions, missing outcome data, outcome measurement, and selective reporting of results. Two authors independently (DV-M and PB) assessed the risk of bias for each included study. Any disagreements were resolved through discussion, and when consensus could not be reached, a third author (PZ) was consulted. Each study was classified as having low risk of bias, some concerns, or high risk of bias at both the domain and overall levels.

### 2.7 Certainty of evidence assessment

Certainty of evidence was assessed using the Grading of Recommendations Assessment, Development and Evaluation (GRADE) (21) approach for each outcome included in the quantitative synthesis. The following domains were considered: risk of bias, inconsistency, indirectness, imprecision, and publication bias. Because the included studies were randomized controlled trials, the certainty of evidence was initially rated as high and then downgraded when concerns were identified in one or more GRADE domains. The overall certainty of evidence was classified as high, moderate, low, or very low.

### 2.8 Data synthesis

A quantitative synthesis was conducted to estimate the pooled effect of curcumin- and ginger-derived interventions on cognitive outcomes. Continuous outcomes were extracted as means and standard deviations at baseline and post-intervention for both the intervention and placebo groups, together with the corresponding sample sizes. For each study, change scores were calculated for both groups. When the standard deviation (SD) of the change was not reported, it was estimated using the baseline and post-intervention SDs assuming a correlation coefficient (r) of 0.7, as recommended in the Cochrane Handbook for systematic reviews (22).

Given the variability in cognitive tests across studies, pooled effect sizes were calculated using the standardized mean difference (SMD) with Hedges’ g correction and corresponding 95% confidence intervals (95% CI). Effect sizes were computed using the inverse-variance method. Considering the expected methodological and clinical heterogeneity among trials (different populations, intervention formulations, doses, and cognitive tests), a random-effects model was applied, with between-study variance (τ^2^) estimated using restricted maximum likelihood (REML).

Statistical heterogeneity was assessed using the I^2^ statistic and Cochran’s Q test. I^2^ values of approximately 25%, 50%, and 75% were interpreted as representing low, moderate, and high heterogeneity, respectively. When substantial heterogeneity was identified, potential sources of heterogeneity were explored qualitatively by comparing key trial-level characteristics, including intervention duration, study population, baseline cognitive status, formulation, dose, and outcome measure. Formal subgroup meta-analyses were not performed when fewer than ten studies were available for a given outcome, because such analyses would be underpowered and potentially misleading. When multiple cognitive outcomes were reported within the same study for a given neuropsychological domain, a single outcome was selected for each meta-analysis to avoid double-counting participants. The selected outcome corresponded to the domain-specific measure entered in the quantitative synthesis. When possible, the study’s prespecified primary endpoint or the most clinically relevant domain-specific measure was prioritized. The specific outcomes selected for each meta-analysis are reported in Supplementary Table 3. To evaluate robustness, sensitivity analyses re-ran the random-effects meta-analyses using the DerSimonian–Laird estimator and Cohen’s d as the effect size metric.

Potential small-study effects/publication bias were assessed by visual inspection of funnel plots and formally tested using Egger’s regression test.

All statistical analyses were performed using Stata (version 19.5, StataCorp, College Station, TX, USA).

## 3 Results

### 3.1 Study selection

**FIGURE 1:**
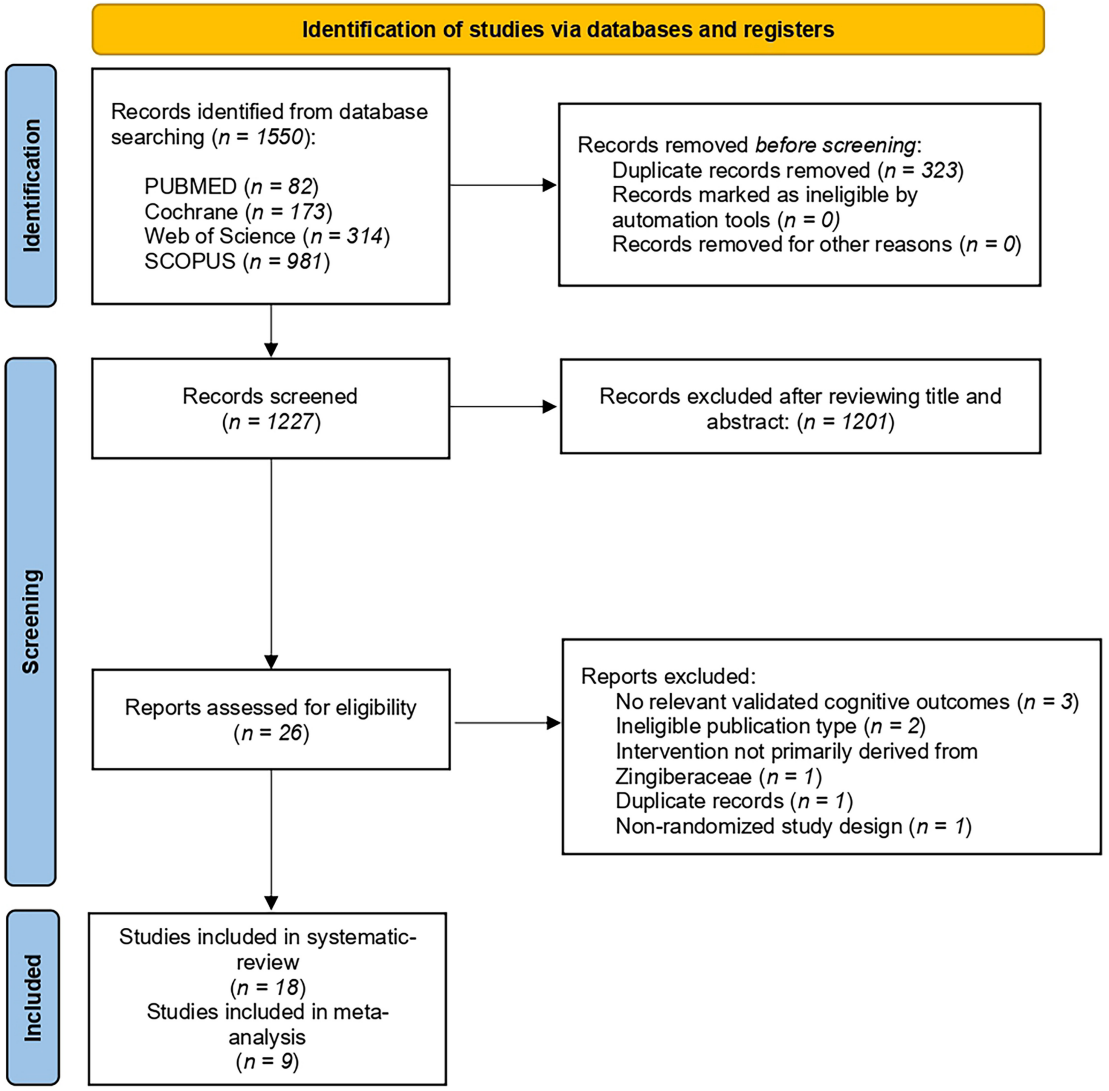
PRISMA flow diagram of the study selection process.

The literature search across the four electronic databases identified 1550 records. After removal of duplicates, 1227 unique records remained for title and abstract screening. Of these, 1201 studies were excluded because they clearly did not meet the predefined eligibility criteria. A total of 26 full-text articles were assessed for eligibility. Following full-text evaluation, 8 studies were excluded for reasons including non-randomized design, absence of relevant cognitive outcomes, interventions not derived from Zingiberaceae compounds, or insufficient reporting of outcome data (Supplementary Table 2). Ultimately, 18 studies met the eligibility criteria and were included in the qualitative synthesis, and 9 studies provided sufficient quantitative data to be included in the meta-analysis. The study selection process is illustrated in Figure 1.

### 3.2 Study characteristics

Eighteen randomized, double-blind, placebo-controlled clinical trials met the inclusion criteria of this review (Table 1), comprising a total of 1355 participants. Sample sizes ranged from 18 participants (23) to 160 participants (24). Across studies, mean participant age ranged from 20.8 ± 1.7 years (25) to 73.5 ± 8.5 years (26), with an overall mean age of 55.3 ± 6.0 years. The proportion of women ranged from 25% (27) to 100% (17, 25, 28), with a mean female representation of 58.6%.

**TABLE 1:** Characteristics of the studies included in the systematic review and meta-analysis.

Study	Country	Design	n	Mean Age (years)	Women (%)	Population	Intervention source/formulation	Dose	Duration and assessment timing	Cognitive outcomes	Main findings
Alimadadi et al., (33)	Iran	RCT-DB-PC	120	32.5	69.8	Adults with major depressive disorder (MDD)	Curcumin capsules	500 mg/day	12 weeks; cognitive outcomes assessed at baseline, week 6, and week 12	Digit Span (forward/backward), Trail Making Test (A/B), Verbal Fluency, Tower of London	Curcumin adjunct therapy significantly improved several executive and memory-related cognitive domains compared with placebo
Badakhshan et al., (32)	Iran	RCT-DB-PC	60	51.7	50.0	Mild cognitive impairment	Zingiber officinale (Zintoma^®^)	750 mg/day	8 weeks; cognitive outcomes assessed at baseline, week 4, and week 8	Global cognition (MMSE, CDR)	Cognitive scores improved compared with placebo
Bahrami et al., (25)	Iran	RCT-TB-PC	124	20.8	100	Women with premenstrual syndrome and dysmenorrhea	Curcumin (C3 Complex^®^ + piperine)	500 mg/day	3 menstrual cycles (10 days/cycle); cognitive outcomes assessed after the intervention period	Memory, inhibitory control, selective attention, total cognitive ability (CAQ)	Significant improvements in memory, inhibitory control, selective attention, and total cognitive scores versus placebo
Cox et al., (12)	Australia	RCT-DB-PC	60	68.5	63.3	Healthy older adults	Solid lipid curcumin formulation (Longvida^®^)	400 mg/day	4 weeks; acute cognitive outcomes assessed at 1 and 3 h after the first dose, with chronic assessment after 4 weeks	Working memory, attention, mood	Significant improvements in cognitive task performance and mood following curcumin supplementation
Cox et al., (13)	Australia	RCT-DB-PC	80	68.1	49.4	Healthy older adults	Lipidated curcumin formulation (Longvida^®^)	400 mg/day	12 weeks; cognitive outcomes assessed at baseline, week 4, and week 12	Working memory, learning ability, mood	Improvements in working memory, mood, and learning-related outcomes
Das et al., (34)	India	RCT-DB-PC	48	64.6	33.3	Adults with moderate dementia due to the onset of Alzheimer’s disease	Curcumin-galactomannan complex (CGM) and standard curcumin (USC)	800 mg/day	24 weeks; cognitive outcomes assessed at baseline and after 6 months	Global cognition (MMSE)	Curcumin formulations improved cognitive scores compared with placebo over 6 months
Gimblet et al., (27)	USA	RCT-DB-PC	88	66.0	25.0	Adults diagnosed with stage 3b or 4 CKD	Curcumin formulation (Longvida^®^)	2000 mg/day	52 weeks; cognitive outcomes assessed at baseline and after 52 weeks	Processing speed, executive function, memory, language (NIH Toolbox Cognition Battery)	Curcumin supplementation did not improve cognitive outcomes compared with placebo
Khanna et al., (23)	India	RCT-DB-PC	18	48.1	33.3	Healthy adults	Curcumin-galactomannan complex (CurQfen^®^)	1000 mg/day	4.3 weeks; cognitive outcomes assessed at baseline and after 30 days	Working memory, verbal recall	Improved memory performance and reduced fatigue
Kuszewski et al., (29)	Australia	RCT-DB-PC	152	65.7	54.0	Overweight/obese, non-depressed adults	Curcumin formulation (Longvida^®^), alone or combined with fish oil	160 mg/day	16 weeks; outcomes assessed at baseline and after 16 weeks	Mood states (POMS), Subjective Memory Complaints (SMCs), Quality of Life (SF-36)	Reduction in subjective memory complaints and improvement in selected wellbeing outcomes
Laksmidewi et al., (28)	Indonesia	RCT-DB-PC	78	48.6	100	Cervical cancer patients undergoing carboplatin–paclitaxel chemotherapy (CICI)	Curcumin extract with intermittent dose escalation	240–400 mg/day (dose-escalation; 14 days on/7 days off)	During chemotherapy cycles; cognitive outcomes assessed pre- and post-therapy	Stroop test (IG score), MoCA-Ina, AFI	Curcumin significantly improved Stroop interference score and MoCA-Ina within group vs baseline; greater Stroop improvement vs placebo; reduced GFAP, IL-6 and isoprostane levels
Lee et al., (37)	Taiwan	RCT-DB-PC	45	73.0	48.9	Community-dwelling adults ≥ 60 years with newly diagnosed untreated pre-diabetes	Turmeric (Curcuma longa) administered as a food-based preparation	1 g	Single acute dose; postprandial cognitive assessment over 6 h	Working Memory	Turmeric improved post-prandial working memory compared with placebo
Nakamura et al., (36)	Japan	RCT-DB-PC	88	47.3	43.8	Healthy adults with eye fatigue and reduced concentration during VDT work	Ginger Extract Powder E (enriched in 6-shogaol)	100 mg/day	12 weeks; outcomes assessed at baseline and week 12 after VDT work	Complex Attention (Cognitrax), Working Memory, Neurocognitive domains; Critical Flicker-Fusion Frequency	Significant improvement in Complex Attention vs placebo after 12 weeks
Rainey-Smith et al., (24)	Australia	RCT-DB-PC	160	66.0	70.8	Community-dwelling older adults	Curcumin formulation (Biocurcumax^®^)	1500 mg/day	52 weeks; cognitive outcomes assessed at baseline, 6 months, and 12 months	Global cognition, verbal memory, executive function	No significant cognitive benefits compared with placebo
Ringman et al., (26)	USA	RCT-DB-PC	36	73.5	63.0	Mild-to-moderate Alzheimer’s disease	Curcumin C3 Complex^®^	2–4 g/day	24 week double-blind phase; open-label extension to 48 weeks	Global cognition, neuropsychiatric symptoms	No improvement in cognitive outcomes; gastrointestinal side effects observed
Saenghong et al., (17)	Thailand	RCT-DB-PC	60	53.4	100	Healthy middle-aged women	Standardized *Zingiber officinale* extract	400 mg/day - 800 mg/day	8 weeks; cognitive and electrophysiological outcomes assessed at baseline, 1 month, and 2 months	Working memory (computerized battery), ERP components (N100, P300)	Significant improvements in working memory, increased N100 and P300 amplitudes, decreased P300 latency (especially 800 mg group)
Santos-Parker et al., (30)	USA	RCT-DB-PC	39	62.0	46.2	Healthy middle-aged and older adults	Curcumin formulation (Longvida^®^)	2000 mg/day	12 weeks; cognitive outcomes assessed at baseline and after 12 weeks	Processing speed, executive function, memory (NIH Toolbox)	Curcumin did not improve cognitive or motor performance
Small et al., (31)	USA	RCT-DB-PC	40	63.0	55.0	Non-demented middle-aged and older adults	Bioavailable curcumin formulation (Theracurmin^®^)	180 mg/day	78 weeks; cognitive and PET outcomes assessed at baseline and endpoint	Verbal and visual memory, attention	Significant improvements in selected verbal and visual memory outcomes and attention; imaging findings suggested reduced amyloid/tau-related signal in some regions
Srivastava et al., (35)	India	RCT-DB-CC	59	22.0	49.0	Healthy young adults with habitual caffeine intake	Alpinia galanga proprietary extract (E-AG-01; EnXtra^®^), alone or with caffeine	300 mg/capsule	Acute, single-dose crossover; outcomes assessed at 1, 3, and 5 h after administration	Mental alertness, sustained attention	Alpinia galanga improved mental alertness versus placebo, and the combination with caffeine improved sustained attention at 3 h while attenuating the caffeine crash

Most studies assessed cognitive outcomes during or at the end of the active intervention period. Post-intervention follow-up after discontinuation of supplementation was generally not reported. AD, Alzheimer’s disease; AFI, Attentional Function Index; CAQ, Cognitive Abilities Questionnaire; CDR, Clinical Dementia Rating; CGM, curcumin-galactomannan complex; CICI, chemotherapy-induced cognitive impairment; CKD, chronic kidney disease; ERP, event-related potentials; GFAP, glial fibrillary acidic protein; IL-6, interleukin-6; MDD, major depressive disorder; MMSE, Mini-Mental State Examination; MoCA-Ina, Indonesian version of the Montreal Cognitive Assessment; NIH, National Institutes of Health; PET, positron emission tomography; POMS, Profile of Mood States; RCT-DB-PC, randomized, double-blind, placebo-controlled trial; RCT-DB-CC, randomized, double-blind, placebo-controlled crossover trial; RCT-TB-PC, randomized, triple-blind, placebo-controlled trial; SF-36, 36-Item Short Form Health Survey; SMCs, Subjective Memory Complaints; USC, standard curcumin; VDT, visual display terminal.

Four studies were conducted in Australia (12, 13, 24, 29), four in the United States (26, 27, 30, 31), three in Iran (25, 32, 33), three in India (23, 34, 35), one in Thailand (17), one in Japan (36), one in Indonesia (28), and one in Taiwan (37).

Eight trials included cognitively healthy middle-aged or older adults (12, 13, 17, 23, 24, 30, 31, 37) while Srivastava et al. (35) enrolled healthy young adults. Regarding baseline cognitive status, some studies included participants with MCI (32, 34), or dementia-level impairment (26). Regarding underlying clinical context or potential contributors to cognitive dysfunction, the included studies involved participants with AD (26), major depressive disorder (33), chronic kidney disease (27), overweight/obese adults (29), women with premenstrual syndrome (25), chemotherapy-induced cognitive impairment (28), and adults with visual display terminal-related attentional complaints (36).

Interventions comprised several Zingiberaceae-derived preparations with substantial differences in source, formulation, dose, dosing schedule, and intended bioavailability. Curcumin-based interventions included enhanced-bioavailability formulations such as Longvida^®^ used by Cox et al.(12), Cox et al. (13), Kuszewski et al. (29), Santos-Parker et al. (30), and Gimblet et al. (27); Theracurmin^®^ used by Small et al. (31); Biocurcumax^®^/BCM-95^®^CG used by Rainey-Smith et al. (24); CurQfen^®^ or curcumin-galactomannan complexes used by Khanna et al. (23) and Das et al. (34); as well as conventional curcumin capsules or extracts used by Bahrami et al. (25), Ringman et al. (26), Laksmidewi et al. (28), and Alimadadi et al. (33). Other interventions included turmeric (Curcuma longa) administered as a food-based preparation in Lee et al. (37), standardized Zingiber officinale preparations in Saenghong et al. (17) and Badakhshan et al. (32), Ginger Extract Powder E enriched in 6-shogaol in Nakamura et al. (36), and an *Alpinia galanga* proprietary extract in Srivastava et al.(35). Daily doses ranged from 100 mg/day for the 6-shogaol-enriched ginger extract used by Nakamura et al. (36) to 4 g/day for Curcumin C3 Complex^®^ used by Ringman et al. (26) in patients with Alzheimer’s disease. Intervention duration also varied widely, from acute single-dose or postprandial assessments with follow-up over several hours in Srivastava et al. (35) and Lee et al. (37), to short-term interventions of approximately 4–12 weeks in Cox et al. (12), Cox et al. (13), Saenghong et al. (17), Khanna et al. (23), Bahrami et al. (25), Santos-Parker et al. (30), Badakhshan et al. (32), Alimadadi et al. (33), and Nakamura et al. (36), medium-term interventions of 16–24 weeks in Kuszewski et al. (29), Ringman et al. (26), and Das et al. (34), and longer interventions of 52–78 weeks in Rainey-Smith et al. (24), Gimblet et al. (26), and Small et al. (31). Most trials assessed cognitive outcomes during or at the end of the active intervention period, whereas post-intervention follow-up after discontinuation of supplementation was generally not reported.

Overall, twelve studies reported significant improvements in at least one cognitive domain compared with placebo (12, 13, 23, 25, 28, 29, 31–36). Improvements were most frequently observed in memory-related outcomes, including working memory, verbal learning, recall, recognition, and related memory measures, as well as in selected attention-related outcomes. In contrast, no significant cognitive benefits were observed in patients with established AD (26), chronic kidney disease (27), or in certain cohorts of healthy older adults (24, 30).

A quantitative synthesis of these outcomes is presented in the subsequent meta-analysis.

### 3.3 Risk of bias assessment

The methodological quality of the included human randomized controlled trials was assessed using the Cochrane Risk of Bias 2 tool. Overall, the risk-of-bias profile was mixed. Several studies were judged as raising some concerns overall, while Rainey-Smith et al., Laksmidewi et al., Ringman et al., and Bahrami et al. were judged as having high risk of bias overall. Cox et al. (13) and Khanna et al. (23) were judged as having low risk of bias overall. Detailed results are presented in Supplementary Figures 1–3.

In the parallel-group trials assessed under ITT assumptions, high risk of bias was identified in Rainey-Smith et al. (24) and Laksmidewi et al. (28), mainly due to missing outcome data. In Rainey-Smith et al. (24), although aggregate cognitive outcome data were available in the published article, 160 participants were randomized and only 96 were included in the final analysis. Participants were excluded because of withdrawals during follow-up and intervention compliance criteria, including a prespecified compliance threshold of ≥70%. Therefore, the efficacy analysis was not based on all randomized participants, and missing endpoint data may have influenced the estimated effect. In Laksmidewi et al. (28), high risk in the missing outcome data domain was related to substantial attrition and incomplete handling of missing endpoint data. The remaining studies in this group were judged as raising some concerns overall, mainly because of insufficiently described allocation concealment, incomplete or unclear reporting of endpoint data, or concerns regarding selection of the reported result.

In the parallel-group trials assessed under PP assumptions, Ringman et al. (26) and Bahrami et al. (25) were judged as having high risk of bias overall. In Ringman et al. (26), the high-risk judgment was mainly driven by missing outcome data, as efficacy analyses were based on completers and withdrawals were more frequent in the curcumin groups. In Bahrami et al. (25), high risk of bias was identified in the randomization process because treatment assignment was based on even or odd numbers in the participant registration list, making allocation predictable before enrollment. Bahrami et al. also raised some concerns regarding missing outcome data and selection of the reported result.

Several other PP-assessed studies raised some concerns overall. These concerns were mainly related to insufficiently described allocation concealment, incomplete outcome data, outcome measurement, or selection of the reported result. Cox et al. (13) and Khanna et al. (23) were judged as having low risk of bias overall. The crossover trial by Srivastava et al. (35) was judged as raising some concerns overall, due to the selection of the reported result domain, while the domains related to randomization, period and carryover effects, deviations from intended interventions, missing outcome data, and outcome measurement were judged as low risk.

### 3.4 Effects of Zingiberaceae-derived interventions on cognitive outcomes

For studies reporting multiple neuropsychological outcomes within the same domain, one outcome per study was selected for each meta-analysis to avoid double-counting participants. The selected outcome measures, time points, and selection rationale for each quantitative synthesis are detailed in Supplementary Table 3.

#### 3.4.1 Memory-related outcomes

**FIGURE 2:**
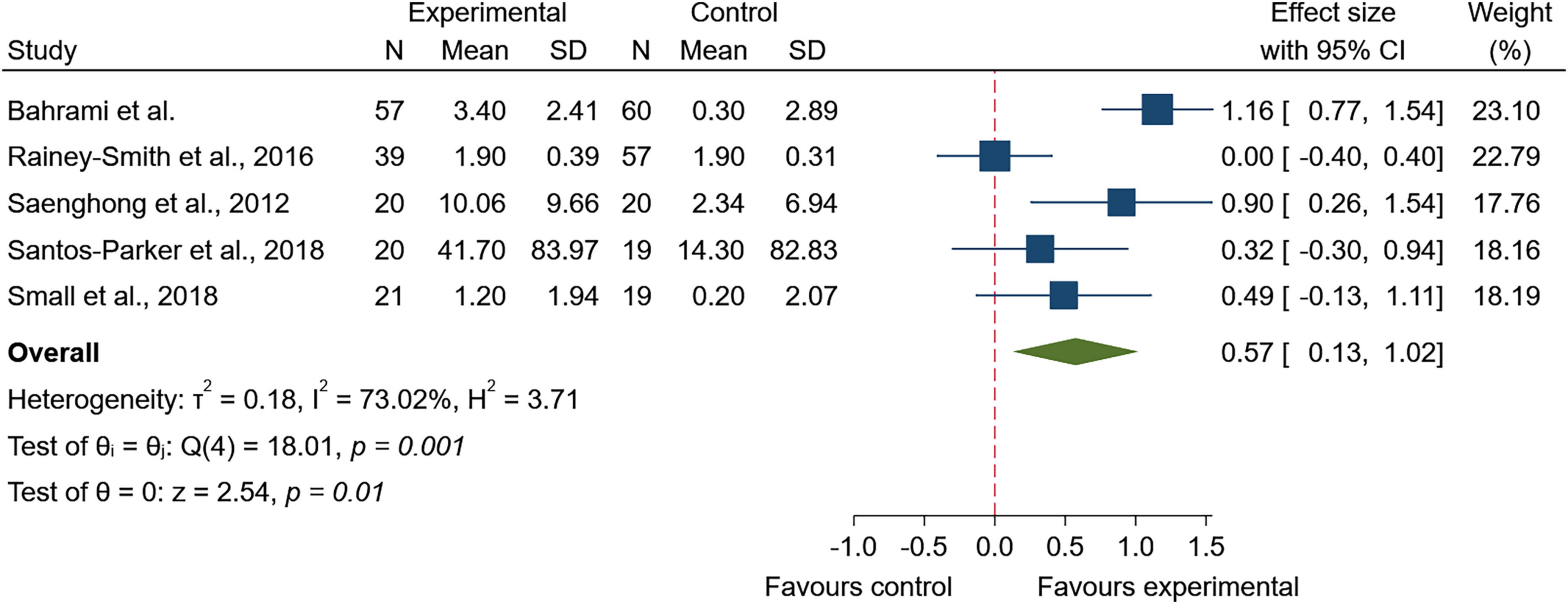
Forest plot of memory-related outcomes following Zingiberaceae-derived interventions versus control.

Five studies were included in the quantitative synthesis for memory-related outcomes. The pooled effect showed a statistically significant improvement in memory-related outcomes in favor of the experimental group (SMD = 0.57; 95% CI: 0.13 to 1.02; *z* = 2.54; *p* < 0.01). Between-study heterogeneity was notable (I^2^ = 73.02%, τ^2^ = 0.18; Figure 2). Given this substantial heterogeneity, potential sources of between-study variability were explored qualitatively. Intervention duration appeared to be one relevant factor. Shorter-term trials, including Saenghong et al. (17) (2 months) and Bahrami et al. (24) (3 menstrual cycles), reported favorable memory-related effects, whereas longer-term trials showed less consistent findings. Rainey-Smith et al. (23) (12 months) did not show a clear sustained benefit on delayed verbal memory, while Small et al. (30) (18 months) reported favorable effects on delayed visual memory. Therefore, treatment duration may have contributed to heterogeneity, although its influence could not be separated from other trial-level differences, including population characteristics, baseline cognitive status, intervention formulation, outcome measure, and risk of bias.

Sensitivity analyses yielded similar results to the primary analysis, with a pooled effect in favor of the experimental group (SMD = 0.58; 95% CI: 0.09 to 1.08; *p* < 0.02). Heterogeneity remained high (I^2^ = 77.75%, τ^2^ = 0.24; Q(4) = 17.98, *p* < 0.001). These results are presented in Supplementary Figure 4A.

Visual inspection of the funnel plot did not suggest marked asymmetry, and Egger’s regression test provided no evidence of small-study effects (β = −0.21, SE = 4.42, z = −0.05, *p* = 0.96; Supplementary Figure 5A).

#### 3.4.2 Executive function and processing speed

**FIGURE 3:**
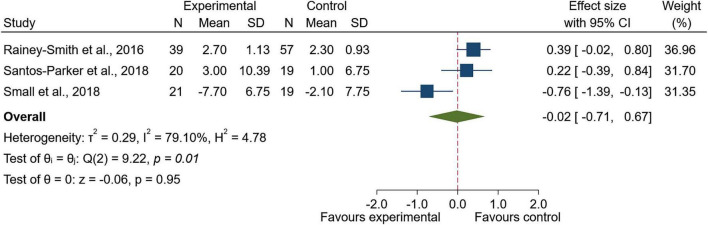
Forest plot of executive function and processing speed outcomes following Zingiberaceae-derived interventions versus control.

Three studies were included in the quantitative synthesis for executive function. The pooled effect did not show a statistically significant difference between groups (SMD = −0.02; 95% CI: −0.71 to 0.67; *z* = −0.06; *p* = 0.95; Figure 3). There was evidence of between-study heterogeneity (I^2^ = 79.10%, τ^2^ = 0.29; Q(2) = 9.22, *p* < 0.01).

Sensitivity analyses were consistent with the primary analysis for executive function, showing no statistically significant between-group difference (SMD = −0.02; 95% CI: −0.71 to 0.67; *z* = −0.07; *p* = 0.95). Evidence of between-study heterogeneity persisted (I^2^ = 78.28%, τ^2^ = 0.29; Q(2) = 9.21, *p* = 0.01). These results are presented in Supplementary Figure 4B. Egger’s regression test showed no evidence of small-study effects (β = −6.32, SE = 7.03, *z* = −0.90, *p* = 0.37; Supplementary Figure 5B), although interpretation is limited by the small number of studies.

#### 3.4.3 Global cognition

**FIGURE 4:**
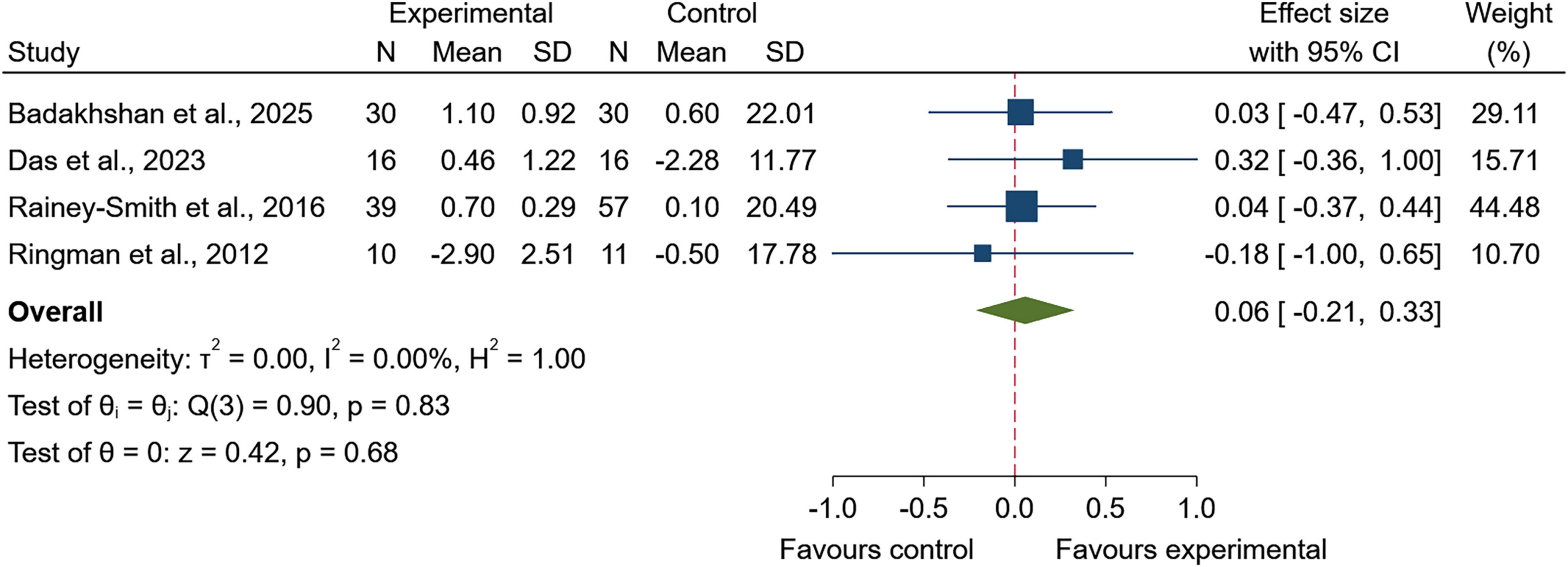
Forest plot of global cognition outcomes following Zingiberaceae-derived interventions versus control.

For global cognition, four studies were pooled. No between-group effect was observed (SMD = 0.06; 95% CI: −0.21 to 0.33; *p* = 0.68; Figure 4), and heterogeneity was not detected (I^2^ = 0.0%, τ^2^ = 0.00; Q(3) = 0.90, *p* = 0.83).

Sensitivity analyses were consistent with the primary analysis for global cognition, showing no evidence of a between-group effect (SMD = 0.06; 95% CI: −0.22 to 0.33; *z* = 0.42; *p* = 0.68), with no detectable heterogeneity (I^2^ = 0.0%, τ^2^ = 0.00; Q(3) = 0.90, *p* = 0.83; Supplementary Figure 4C). Funnel plot inspection did not suggest marked asymmetry (Supplementary Figure 5C), and Egger’s regression test provided no evidence of small-study effects (β = 0.01, SE = 1.91, *z* = 0.01, *p* = 0.99).

#### 3.4.4 Attention or inhibitory control

**FIGURE 5:**
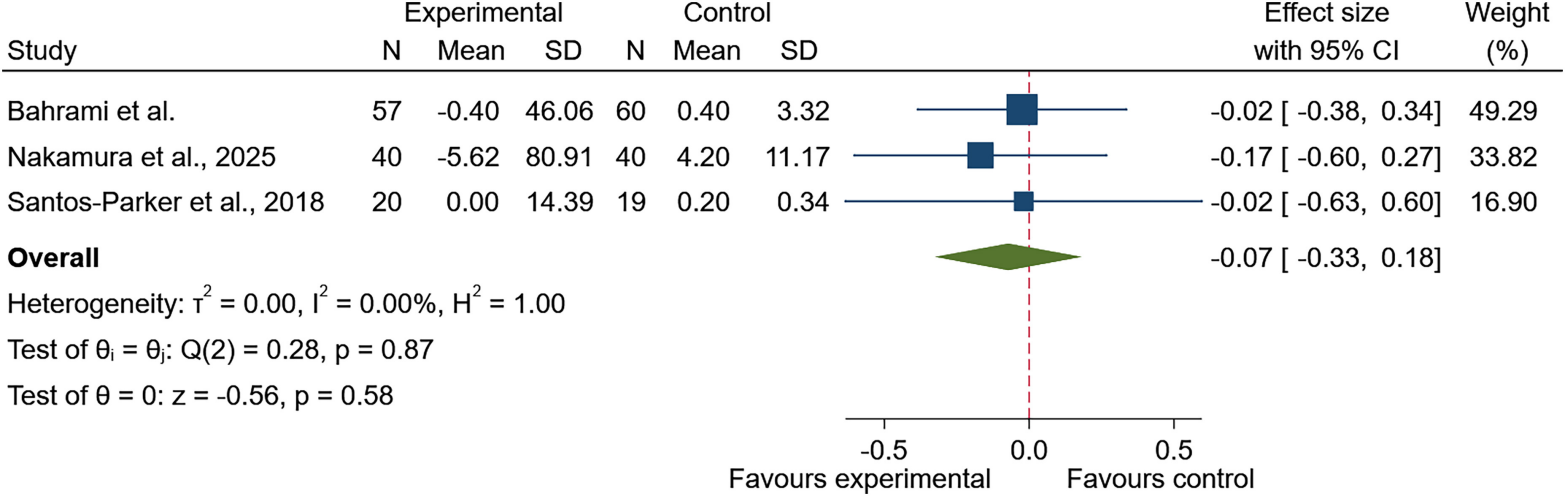
Forest plot of attention or inhibitory control outcomes following Zingiberaceae-derived interventions versus control.

For attention or inhibitory control, evidence from three studies did not indicate a difference between the experimental and control groups (SMD = −0.07; 95% CI: −0.33 to 0.18; *p* = 0.58; Figure 5). Between-study variability was minimal (I^2^ = 0.0%, τ^2^ = 0.00; Q(2) = 0.28, *p* = 0.87). These findings were unchanged in sensitivity analyses (SMD = −0.07; 95% CI: −0.33 to 0.18; *p* = 0.58; Supplementary Figure 4D). Funnel plot inspection did not suggest asymmetry (Supplementary Figure 5D), and Egger’s test showed no evidence of small-study effects (β = −0.03, SE = 2.79, *z* = −0.01, *p* = 0.99).

#### 3.4.5 Safety and tolerability findings

Safety and tolerability reporting was variable across the included trials, but when these outcomes were explicitly assessed, Zingiberaceae-derived interventions were generally described as acceptable or well tolerated. Ringman et al. (26) reported frequent adverse events in both placebo- and curcumin-treated participants, with several withdrawals in the curcumin arms due to gastrointestinal complaints and difficulty swallowing, although no serious adverse events were observed. Gimblet et al. (27) similarly found no statistically significant between-group differences in overall adverse events, nausea, vomiting, or dizziness, and described curcumin supplementation as safe and well tolerated, despite a numerically higher frequency of abdominal pain in the curcumin group. Laksmidewi et al. (28) reported that dropout, mortality, and adverse drug responses were broadly comparable between groups, with palpitations, paresthesias, and diarrhea recorded in the curcumin arm. Small et al. (31) also described gastrointestinal complaints, with transient abdominal pain, gastritis, or nausea occurring in both groups but numerically more often with curcumin. Rainey-Smith et al. (24) considered the formulation generally well tolerated, but gastrointestinal complaints accounted for most suspected adverse events and contributed to participant withdrawal. In ginger-based trials, Nakamura et al. (36) reported no adverse events or safety issues under the study conditions, whereas Badakhshan et al. (32) found no significant between-group difference in side effects, with isolated heartburn reported in one participant per arm. Bahrami et al. (25) also stated that adverse reactions were monitored and described curcumin as safe and well tolerable. Overall, the available evidence suggests that these interventions are often reasonably well tolerated in the short to medium term, but the marked inconsistency in adverse-event ascertainment and reporting across trials limits firm conclusions, and gastrointestinal intolerance appears to be the most recurrent adverse effect, particularly in some curcumin studies.

#### 3.4.6 Certainty of evidence

The certainty of evidence was assessed using the GRADE approach for the four outcomes included in the quantitative synthesis. Overall, the certainty of evidence ranged from low to very low. For episodic memory, the certainty of evidence was rated as very low because of serious concerns related to risk of bias, substantial inconsistency, and imprecision. Although the pooled effect favored Zingiberaceae-derived interventions, this finding should therefore be interpreted as preliminary.

For executive function and processing speed, the certainty of evidence was rated as very low due to risk of bias, substantial inconsistency, and imprecision, with confidence intervals compatible with benefit, no effect, or harm. For global cognition, the certainty of evidence was rated as low, mainly because of risk of bias and imprecision, although statistical heterogeneity was not detected. For attention or inhibitory control, the certainty of evidence was rated as very low because of risk of bias, indirectness across attention-related constructs, and imprecision. The complete GRADE Summary of Findings is presented in Supplementary Table 4.

## 4 Discussion

The present systematic review and meta-analysis synthesized randomized controlled trials evaluating whether Zingiberaceae-derived interventions improve cognitive outcomes in adults. The main quantitative finding was a statistically significant pooled effect favoring the intervention for memory-related outcomes, whereas no pooled benefits were observed for executive function/processing speed, global cognition, or attention/inhibitory control. This pattern is consistent with the possibility that any cognitive effects are not broad or generalized, but rather more likely to emerge in selected domains and under specific study conditions. Previous quantitative syntheses have largely focused on curcumin alone, and their findings likewise suggest that benefits tend to cluster around selected domains rather than being reflected in uniformly improved cognition. For example, Zhu et al. (38) concluded that curcumin appeared more effective in older adults than in patients with AD or schizophrenia, whereas Tsai et al. (39) reported that the cognitive effects of curcumin varied across domains and were not consistently captured by global cognitive outcomes. More recent evidence syntheses, including those by Wang et al. (40) and Yu et al. (14) have also examined curcumin supplementation in relation to cognition and cognitive aging. In this context, the present review both aligns with and extends previous work by moving beyond a curcumin-only framework to examine the broader family of Zingiberaceae-derived interventions, including turmeric- and ginger-based preparations.

This domain-specific pattern is biologically and clinically plausible, but it should be interpreted within the distinction between cognitive severity and underlying etiology. Episodic memory is one of the cognitive domains most sensitive to early clinical change in several aging-related conditions, including but not limited to AD. Episodic memory impairment is a characteristic feature of early AD-related decline (41), but memory complaints and measurable memory deficits may also occur in individuals with subjective cognitive complaints, MCI of non-AD or mixed etiology, cerebrovascular disease, metabolic dysfunction, mood disorders, and other clinical contexts. Therefore, the significant pooled effect observed for memory-related outcomes should not be interpreted as evidence of efficacy specifically in AD pathology. Rather, it suggests that memory-related measures may be more sensitive than broader global cognitive screening tools for detecting possible domain-specific effects of Zingiberaceae-derived interventions across heterogeneous adult populations (42).

This interpretation is also consistent with the characteristics of the individual trials included in our review. Several of the positive studies reported improvements in memory measures, including working memory, verbal learning, recall, recognition, or related memory outcomes, even when other domains did not clearly change, including trials by Cox et al. (12, 13), Khanna et al. (23), Lee et al. (37), Saenghong et al. (17), and Small et al. (31) using Longvida^®^, Theracurmin^®^, CurQfen^®^, turmeric, or ginger-derived preparations. At the same time, studies that relied on broader global measures often yielded null or less consistent findings, as illustrated by Ringman et al. (26), Rainey-Smith et al. (24), and Gimblet et al. (27). This is important because the MMSE has repeatedly been criticized for poor sensitivity to subtle cognitive deficits and for limited utility in detecting early or mild impairment. Evidence from both narrative and diagnostic studies suggests that the MMSE may miss mild cognitive impairment and early-stage decline, whereas the MoCA is generally more sensitive to subtle changes (43–45). Therefore, the lack of effect observed for global cognition in the present analysis should not necessarily be interpreted as evidence that these interventions are globally ineffective; rather, it may reflect the limited responsiveness of some global screening tools and the fact that subtle, domain-specific gains are more likely to emerge on targeted neuropsychological measures than on brief cognitive screeners.

The interpretation of the memory-related findings should also consider the risk-of-bias profile of the contributing studies. Some trials reporting favorable effects on memory-related outcomes had methodological concerns related to allocation concealment, missing outcome data, or selection of the reported result. In particular, Bahrami et al. (25) used a predictable allocation procedure based on even or odd registration numbers, and Saenghong et al. (17) did not clearly describe allocation concealment and raised some concerns regarding endpoint data reporting. These limitations are especially relevant because both studies reported positive cognitive findings. Therefore, the significant pooled effect for memory-related outcomes should be interpreted cautiously and should not be considered definitive evidence of efficacy.

A key contribution of the present review is that it expands the literature beyond curcumin-only approaches. In addition to curcumin formulations, our review included turmeric and ginger-derived interventions, thereby reflecting the broader Zingiberaceae family and better capturing the diversity of plant-derived compounds that may influence cognition. This broader scope is clinically relevant, but it also likely contributed to the observed heterogeneity. The included trials used multiple formulations, including Longvida^®^, Theracurmin^®^, Biocurcumax^®^, CurQfen^®^, curcumin-galactomannan complexes, conventional curcumin capsules, turmeric in food form, and ginger extracts enriched in compounds such as 6-shogaol, which have been linked to antioxidant, anti-inflammatory, and potentially neuroprotective effects, as well as possible modulation of cholinergic and monoaminergic pathways in the brain (46). As noted by Francis et al. (15) the clinical literature on curcumin remains difficult to interpret because of substantial variation in formulation, dose, population, and outcome selection. This concern is reinforced by pharmacokinetic studies showing marked differences in systemic exposure across commercial curcumin products (47, 48) as well as by methodological work showing that bioavailability is frequently acknowledged but insufficiently addressed in evidence syntheses (48).

Intervention duration and timing of cognitive assessment may also have contributed to the heterogeneity observed across cognitive domains. Acute or very short-term studies are more likely to capture transient effects on alertness, attention, postprandial cognitive performance, or working memory, particularly when outcomes are assessed within hours of administration. This was the case in Lee et al. (37), who evaluated postprandial working memory over a 6-h period after turmeric intake, and in Srivastava et al. (35), who assessed mental alertness and sustained attention at 1, 3, and 5 h after acute administration of Alpinia galanga, caffeine, or their combination. By contrast, memory-related outcomes linked to neuroplasticity, inflammatory modulation, mitochondrial function, or amyloid- and tau-related pathways may require longer exposure periods before measurable effects emerge. For example, Cox et al. (12) assessed both acute effects at 1 and 3 h and chronic effects after 4 weeks of Longvida^®^ supplementation, whereas Small et al. (31) evaluated Theracurmin^®^ over 18 months and reported improvements in verbal and visual memory together with imaging changes in amyloid- and tau-related signal. However, longer duration did not guarantee efficacy, as Rainey-Smith et al. (24), using Biocurcumax^®^/BCM-95^®^CG for 12 months, and Gimblet et al. (27), using Longvida^®^ for 52 weeks in chronic kidney disease, did not produce consistent cognitive benefits. Therefore, duration should not be interpreted in isolation, but rather together with formulation bioavailability, dose, baseline cognitive status, underlying pathophysiology, and cognitive test sensitivity. Importantly, most trials did not include a post-discontinuation follow-up period, limiting conclusions regarding the persistence of cognitive effects after supplementation ceased. Overall, although intervention duration may partly explain the variability in memory-related effects, the current evidence is insufficient to define an optimal intervention length.

Population heterogeneity is another likely explanation for the mixed findings, and this heterogeneity should be considered at two distinct levels: baseline cognitive status and underlying clinical context or potential etiology. The studies included cognitively healthy middle-aged and older adults (12, 13, 17, 30, 31), individuals with mild cognitive impairment (32, 34), AD (26), chronic kidney disease (27), major depressive disorder (33), chemotherapy-induced cognitive impairment (28), overweight/obesity (29), prediabetes (37), premenstrual syndrome (25), and visual display terminal-related attentional complaints (36). Importantly, mild cognitive impairment describes the severity or stage of cognitive impairment, whereas AD, chronic kidney disease, major depressive disorder, chemotherapy-related cognitive impairment, metabolic dysfunction, and other clinical conditions refer to possible underlying etiologies or contributors to cognitive dysfunction. These dimensions should therefore not be conflated.

These populations differ substantially in the underlying drivers of cognitive dysfunction. Cognitive inefficiency associated with aging or prodromal neurodegeneration is not equivalent to the cognitive burden associated with depression, chemotherapy, metabolic dysfunction, systemic inflammation, or chronic kidney disease. It is therefore unsurprising that the trials did not converge neatly on the same pattern of response. In fact, the literature suggests that curcumin-related interventions may be more promising in settings characterized by low-grade inflammation, oxidative stress, or early functional decline than in advanced neurodegenerative disease. Zhu et al. (38) explicitly reported that curcumin appeared more effective in elderly populations than in AD or schizophrenia, which supports the idea that clinical context materially influences response. This may help explain why trials in AD and chronic kidney disease were negative in our review. Ringman et al. (26) found no cognitive benefit in mild-to-moderate AD, despite relatively high doses of curcumin, and gastrointestinal side effects were also observed. A plausible explanation is that once neurodegeneration is established, antioxidant or anti-inflammatory interventions may be less able to produce measurable cognitive recovery, especially if the formulation does not achieve sufficient brain exposure. Similarly, the negative findings reported by Gimblet et al. (27) in chronic kidney disease may reflect the complex and multifactorial basis of cognitive dysfunction in that population, in which vascular injury, uremic toxin accumulation, oxidative stress, inflammation, and other systemic contributors related to kidney dysfunction have all been implicated (49).

The lack of a pooled effect for executive function/processing speed is also worth discussing carefully. Executive outcomes were heterogeneous both conceptually and statistically in the present review. Some trials suggested improvement in selected tasks, while others found null effects or even point estimates favoring control. This variability may partly reflect differences in the sensitivity and ecological validity of the cognitive tests used. Executive function is a broad construct that encompasses inhibition, set-shifting, working memory manipulation, reasoning, and processing efficiency, and different batteries capture these components differently. Therefore, the absence of a significant pooled effect may reflect methodological dispersion as much as true inefficacy. Tsai et al. (39) made a similar observation in their meta-analysis, reporting that the effect of curcumin differed across individual domains and that the literature remained inconclusive overall.

A related point concerns attention and inhibitory control, for which no significant pooled effect was detected. Although certain individual studies in our review reported improvements in selected attention-related outcomes, such as Stroop interference or complex attention, the overall quantitative signal remained null. This suggests that attention-related benefits may be context-dependent rather than generalizable. For example, the positive findings in visual display terminal-related attentional complaints or chemotherapy-induced cognitive impairment may reflect specific biological or symptomatic contexts in which attentional dysfunction is especially modifiable. In the case of cancer-related cognitive impairment, mechanisms such as neuroinflammation, oxidative stress, mitochondrial dysfunction, and impaired neuroplasticity have been proposed (50), whereas prolonged exposure to visual display terminals has been associated with visual fatigue, mental fatigue, and reduced sustained attention (51). That possibility is consistent with the broader literature on nutraceuticals and cognition, where effects are often stronger in symptom-defined or biologically stressed populations than in unselected community samples.

The current findings should also be interpreted in light of the proposed mechanisms of action of Zingiberaceae compounds. Curcumin and ginger-derived constituents have repeatedly been linked to reduced oxidative stress, downregulation of inflammatory signaling pathways such as nuclear factor kappa B (NF-κB), and modulation of neurotrophic, vascular, and mitochondrial processes. In addition to these broad mechanisms, recent evidence suggests that curcumin may influence antioxidant defense pathways, including nuclear factor erythroid 2-related factor 2 (Nrf2)-related signaling, as well as processes involved in neuroplasticity, synaptic remodeling, mitochondrial function, and brain-derived neurotrophic factor (BDNF)-related signaling (52, 53). Ginger-derived compounds, particularly gingerols and shogaols, have also been associated with antioxidant, anti-inflammatory, and immunomodulatory effects, and may modulate pathways relevant to neuronal function and cognitive performance (54, 55). Francis et al. (15) reviewed clinical and biomarker evidence suggesting that curcumin may influence inflammatory markers alongside neurocognitive outcomes, while broader formulation reviews have highlighted the mechanistic rationale for central nervous system effects once sufficient systemic exposure is achieved. These mechanisms provide a credible biological basis for the improvements seen in some trials, particularly in memory-related outcomes (48). However, they do not guarantee uniform clinical efficacy, especially when formulations differ in bioavailability and when the enrolled populations have distinct pathophysiological drivers of impairment. This is particularly relevant for curcumin, given its poor oral bioavailability and the marked pharmacokinetic variability observed across conventional and enhanced-bioavailability formulations (56). Therefore, antioxidant, anti-inflammatory, neurotransmitter-related, and brain-signaling mechanisms should be interpreted in conjunction with formulation characteristics, dose, duration, baseline cognitive status, and the sensitivity of the cognitive outcome assessed.

An additional methodological issue concerns the interpretation of small-study effects and publication bias. In our meta-analyses, visual inspection of funnel plots and Egger’s regression tests did not suggest marked asymmetry. However, these findings should be interpreted very cautiously. As emphasized in the Cochrane Handbook (22), statistical methods for detecting funnel plot asymmetry are of limited value when only a small number of studies are available, and results can be unstable or uninformative in such settings. Because each of our quantitative syntheses included fewer than ten studies, the absence of statistical evidence for small-study effects should not be taken as strong evidence that publication bias is absent (15, 38). The risk-of-bias profile of the included studies was mixed. Although some trials were judged at low risk of bias, most raised concerns in at least one domain, and three studies were judged as having high risk of bias overall. The most relevant concerns involved insufficiently described allocation concealment, predictable allocation procedures, missing outcome data, completer-based or per-protocol analyses, and selection of the reported result. These issues are particularly important in cognitive intervention trials, where small sample sizes, repeated testing, practice effects, and attrition may influence observed effects. Accordingly, the memory-related findings should be interpreted with caution, especially because most studies reporting positive findings in memory-related outcomes were judged as having some concerns or high risk of bias.

Overall, our findings support a nuanced interpretation. The present evidence does not suggest that Zingiberaceae-derived interventions improve all aspects of cognition in adults. Instead, it suggests that benefits may be selective, with memory-related outcomes emerging as the most promising target, while effects on global cognition and other domains remain uncertain. This conclusion is more aligned with the available literature than a simplistic positive-or-negative verdict. The field now needs larger, adequately powered randomized trials with clearer primary outcomes, standardized cognitive batteries, careful baseline phenotyping, and more explicit consideration of formulation-related bioavailability. Stratification by intervention type, dose, duration, and baseline cognitive status will be particularly important if future studies are to determine whether the observed memory benefit reflects a true class effect, a formulation effect, or an interaction between intervention type and clinical phenotype (48, 57).

The GRADE assessment further supports this cautious interpretation. The certainty of evidence was low or very low across all outcomes included in the quantitative synthesis. Therefore, the observed memory-related effect should be considered preliminary and hypothesis-generating rather than definitive evidence of efficacy.

### 4.1 Strengths and limitations

This review has several strengths. First, it focused exclusively on randomized, placebo-controlled clinical trials. Second, unlike previous reviews centered mainly on curcumin alone, it adopted a broader Zingiberaceae perspective, allowing a more inclusive interpretation of the available evidence. Third, the analyses were conducted by cognitive domain, which is particularly important in this field because global cognitive outcomes may obscure selective domain-specific effects. Finally, sensitivity analyses were consistent with the main findings, supporting the robustness of the overall pattern observed.

Several limitations should also be acknowledged. The number of studies available for each quantitative synthesis was small, limiting statistical power and preventing more detailed subgroup meta-analyses or meta-regression. Although potential sources of heterogeneity, including intervention duration, were explored qualitatively, the small number of studies per outcome precluded further quantitative exploration. Heterogeneity was substantial for some outcomes, likely due to differences in botanical source, preparation type, formulation bioavailability, dose, dosing schedule, intervention duration, timing of cognitive assessment, population type, baseline cognitive status, and cognitive assessment methods. Moreover, most trials did not include post-intervention follow-up after discontinuation of supplementation, limiting conclusions regarding the persistence of cognitive effects. In addition, the interventions included under the Zingiberaceae umbrella were pharmacologically diverse, so the pooled estimates should not be interpreted as evidence that all family-derived compounds have equivalent cognitive effects. Some studies also involved highly specific clinical contexts, such as chemotherapy-related cognitive impairment (28) or visual display terminal-associated attentional complaints (36) which may limit generalizability to broader populations. Finally, the current clinical evidence remains much stronger for curcumin than for ginger-derived compounds, so conclusions regarding the family as a whole should still be interpreted cautiously. Another important limitation is the risk of bias identified in some trials contributing positive cognitive findings. Predictable allocation procedures insufficiently described allocation concealment, incomplete or unclear reporting of endpoint data, attrition, and analyses based on completers or per-protocol samples may exaggerate treatment effects, particularly in small trials with cognitive outcomes that may be sensitive to practice effects, expectancy, or attrition. These concerns contributed to downgrading the certainty of the evidence and support a cautious interpretation of the observed memory-related effect.

Taken together, these findings support cautious interpretation and underscore the need for larger, more homogeneous, adequately powered trials using clearly characterized formulations and sensitive domain-specific cognitive endpoints.

## 5 Conclusion

This systematic review and meta-analysis suggests that Zingiberaceae-derived interventions may improve memory-related outcomes, but the evidence is very uncertain. Therefore, this effect should be interpreted cautiously because the certainty of the evidence is limited by the small number of studies, substantial heterogeneity, variability in populations and formulations, and methodological concerns in some trials reporting positive cognitive effects, particularly regarding allocation concealment, missing outcome data, and selection of the reported result. No consistent pooled effects were identified for executive function and processing speed, global cognition, or attention/inhibitory control. Larger, rigorously designed trials with adequate allocation concealment, transparent reporting of participant flow and attrition, standardized cognitive endpoints, clearly prespecified outcomes, and well-characterized bioavailable formulations are needed.

## Data Availability

The original contributions presented in the study are included in the article/Supplementary material, further inquiries can be directed to the corresponding author.

## Supplementary material

Supplementary Figure 1 Cochrane Risk of Bias 2 (RoB 2) assessment for parallel-group randomized controlled trials assessed under intention-to-treat (ITT) assumptions.

Supplementary Figure 2 Cochrane Risk of Bias 2 (RoB 2) assessment for parallel-group randomized controlled trials assessed under per-protocol (PP) assumptions.

Supplementary Figure 3 Cochrane Risk of Bias 2 (RoB 2) assessment for the crossover randomized controlled trial.

Supplementary Figure 4 Sensitivity analysis forest plots for the cognitive outcomes included in the meta-analysis. **(A)** Episodic memory; **(B)** executive function/processing speed; **(C)** global cognition; **(D)** attention or inhibitory control.

Supplementary Figure 5 Funnel plots with Egger’s regression tests for the same cognitive outcomes. **(A)** Episodic memory; **(B)** executive function; **(C)** global cognition; **(D)** attention or inhibitory control.
