# Supplementary material for: Effects of Zingiberaceae-derived interventions on memory-related and other cognitive outcomes in adults: a systematic review and meta-analysis
Source: Front Nutr. 2026 May 11;13:1834167. doi: 10.3389/fnut.2026.1834167 (PMC13198985; doi:10.3389/fnut.2026.1834167)
Supplement: Supplementary file 1 [file Table_1.docx]

**Table S1**. Search strategy.

| **PubMed/Medline**: March 5, 2026 (82) |
| --- |
| (("Zingiberaceae"[Title/Abstract] OR "Curcuma longa"[Title/Abstract] OR turmeric[Title/Abstract] OR curcumin[Title/Abstract] OR curcuminoids[Title/Abstract] OR "Zingiber officinale"[Title/Abstract] OR ginger[Title/Abstract] OR "ginger extract"[Title/Abstract] OR gingerols[Title/Abstract] OR shogaols[Title/Abstract] OR "Zingiber purpureum"[Title/Abstract] OR "Java ginger"[Title/Abstract]) AND (memory[Title/Abstract] OR "memory performance"[Title/Abstract] OR cognition[Title/Abstract] OR "cognitive function"[Title/Abstract] OR "cognitive decline"[Title/Abstract] OR "cognitive impairment"[Title/Abstract] OR "mild cognitive impairment"[Title/Abstract] OR MCI[Title/Abstract] OR dementia[Title/Abstract] OR "Alzheimer disease"[Title/Abstract] OR "age-related cognitive decline"[Title/Abstract]) AND (randomized[Title/Abstract] OR randomised[Title/Abstract] OR placebo[Title/Abstract] OR "clinical trial"[Title/Abstract] OR trial[Title/Abstract] OR "double blind"[Title/Abstract])) |
| **Cochrane**: March 5, 2026 (173) |
| (Zingiberaceae OR "Curcuma longa" OR turmeric OR curcumin OR curcuminoids OR "Zingiber officinale" OR ginger OR "ginger extract" OR gingerols OR shogaols OR "Zingiber purpureum" OR "Java ginger") AND (memory OR "memory performance" OR cognition OR "cognitive function" OR "cognitive decline" OR "cognitive impairment" OR "mild cognitive impairment" OR MCI OR dementia OR "Alzheimer disease" OR "age-related cognitive decline") |
| **Scopus**: March 5, 2026 (981) |
| TITLE-ABS-KEY (("Zingiberaceae" OR "Curcuma longa" OR turmeric OR curcumin OR curcuminoids OR "Zingiber officinale" OR ginger OR "ginger extract" OR gingerols OR shogaols OR "Zingiber purpureum" OR "Java ginger") AND (memory OR "memory performance" OR cognition OR "cognitive function" OR "cognitive decline" OR "cognitive impairment" OR "mild cognitive impairment" OR MCI OR dementia OR "Alzheimer disease" OR "age-related cognitive decline") AND (randomized OR randomised OR placebo OR "clinical trial" OR trial OR "double blind")) |
| **Web of Science**: March 5, 2026 (314) |
| TS=(("Zingiberaceae" OR "Curcuma longa" OR turmeric OR curcumin OR curcuminoids OR "Zingiber officinale" OR ginger OR "ginger extract" OR gingerols OR shogaols OR "Zingiber purpureum" OR "Java ginger") AND (memory OR "memory performance" OR cognition OR "cognitive function" OR "cognitive decline" OR "cognitive impairment" OR "mild cognitive impairment" OR MCI OR dementia OR "Alzheimer disease" OR "age-related cognitive decline") AND (randomized OR randomised OR placebo OR "clinical trial" OR trial OR "double blind")) |
| **Total**: 1550 |
