# Supplementary material for: Effects of Zingiberaceae-derived interventions on memory-related and other cognitive outcomes in adults: a systematic review and meta-analysis
Source: Front Nutr. 2026 May 11;13:1834167. doi: 10.3389/fnut.2026.1834167 (PMC13198985; doi:10.3389/fnut.2026.1834167)
Supplement: Supplementary file 2 [file Table_2.docx]

**Table S2.** Excluded studies and reasons for exclusion.

| DiSilvestro RA, Joseph E, Zhao S, Bomser J. Diverse effects of a low dose supplement of lipidated curcumin in healthy middle aged people. Nutr J. 2012;11:79. |
| --- |
| Reason for exclusion: **No relevant validated cognitive outcomes.** The study evaluated biochemical and wellness-related markers, such as triglycerides, nitric oxide, sICAM, salivary amylase, and antioxidant-related measures, but did not assess cognitive outcomes using validated neuropsychological instruments relevant to the predefined review domains. |
| Dost FS, et al. Theracurmin Supplementation May be a Therapeutic Option for Older Patients with Alzheimer’s Disease: A 6-Month Retrospective Follow-Up Study. Curr Alzheimer Res. 2021;18:1087–1092. |
| Reason for exclusion: **Non-randomized study design.** This was a retrospective follow-up study based on medical record review rather than a randomized controlled trial, and therefore did not meet the eligibility criteria of the review. |
| Wang H-C, Liu N-Y, Zhang S, et al. Clinical Experience in Treatment of Alzheimer’s Disease with Jiannao Yizhi Formula and Routine Western Medicine. Chin J Integr Med. 2020;26(3):212–218. |
| Reason for exclusion: **Intervention not primarily derived from Zingiberaceae.** Although cognitive outcomes were assessed, the intervention was a multicomponent traditional Chinese medicine formula composed of several herbal ingredients, and the effects could not be attributed specifically to a Zingiberaceae-derived intervention. |
| Sha Z, Zhao Z, Li N, et al. Efficacy and safety of Yi Shen Fang granules in elderly people with MCI: study protocol for a multicentre, randomized, double-blind, parallel-group, controlled trial. BMC Complement Med Ther. 2023;23:101. |
| Reason for exclusion: **Ineligible publication type.** This article was a study protocol and did not report completed trial results eligible for qualitative or quantitative synthesis. In addition, the intervention was a multicomponent herbal formula rather than a clearly attributable Zingiberaceae-derived preparation. |
| Wang BQ, Mei J, Liu L, et al. Exploratory study on the safety and effectiveness of Yizhi Qingxin Decoction (capsules) in the treatment of hypertension in the elderly with mild cognitive impairment. Medicine (Baltimore). 2020;99(27):e20789. |
| Reason for exclusion: **Ineligible publication type.** This article was published as a study protocol clinical trial and did not provide completed outcome data for inclusion in the review. |
| Ashraf H, Heydari M, Shams M, et al. Efficacy of Ginger Supplementation in Relieving Persistent Hypothyroid Symptoms in Patients with Controlled Primary Hypothyroidism: A Pilot Randomized, Double-Blind, Placebo-Controlled Clinical Trial. Evid Based Complement Alternat Med. 2022;2022:5456855. |
| Reason for exclusion: **No relevant validated cognitive outcomes.** Although some symptom domains included memory loss and concentration disturbance, the primary outcome was symptom relief assessed with the Thyroid Symptom Rating Questionnaire rather than validated cognitive testing aligned with the predefined neurocognitive domains of the review. |
| Kuszewski JC, Howe PRC, Wong RHX. An Exploratory Analysis of Changes in Mental Wellbeing Following Curcumin and Fish Oil Supplementation in Middle-Aged and Older Adults. Nutrients. 2020;12:2902. |
| Reason for exclusion: **Duplicate publication (overlapping sample).** This article was a secondary/exploratory report from the same 16-week randomized, double-blind, placebo-controlled trial population already represented elsewhere in the review; therefore, it was excluded to avoid double counting of participants. |
| Thota RN, Rosato JI, Dias CB, Burrows TL, Martins RN, Garg ML. Dietary Supplementation with Curcumin Reduce Circulating Levels of Glycogen Synthase Kinase-3β and Islet Amyloid Polypeptide in Adults with High Risk of Type 2 Diabetes and Alzheimer's Disease. Nutrients. 2020;12(4):1032. |
| Reason for exclusion: **No relevant validated cognitive outcomes.** The study primarily evaluated circulating biomarkers related to glycogen synthase kinase-3β, islet amyloid polypeptide, insulin resistance, and metabolic risk, rather than validated neuropsychological outcomes aligned with the predefined cognitive domains of the review. |
