# Supplementary material for: Effects of Zingiberaceae-derived interventions on memory-related and other cognitive outcomes in adults: a systematic review and meta-analysis
Source: Front Nutr. 2026 May 11;13:1834167. doi: 10.3389/fnut.2026.1834167 (PMC13198985; doi:10.3389/fnut.2026.1834167)
Supplement: Supplementary file 3 [file Table_3.docx]

Table S3. Neuropsychological outcomes selected for each meta-analysis

| Meta-analysis/domain | Study | Selected neuropsychological outcome | Time point/contrast selected | Reason for selection | Other relevant cognitive outcomes reported |
| --- | --- | --- | --- | --- | --- |
| Episodic memory | Small et al., 2018 | Brief Visual Memory Test - Delay | 18 months | Selected as the delayed visual memory outcome entered in the episodic memory meta-analysis. | Buschke Selective Reminding Test, Brief Visual Memory Test – Recall, Trail Making Test Part A. |
|  | Rainey-Smith et al., 2016 | Rey Auditory Verbal Learning Test delayed recall | 12 months | Selected as the delayed verbal memory outcome entered in the episodic memory meta-analysis. | MoCA, RAVLT list learning, RAVLT short-term recall, Wechsler Digit Symbol Scale, COWAT, cognitive composite scores. |
|  | Santos-Parker et al., 2018 | NIH Toolbox Picture Sequence Memory Test | 12 weeks | Selected as the episodic memory measure from the NIH Toolbox battery. | NIH Toolbox Pattern Comparison Processing Speed, Flanker Inhibitory Control and Attention, List Sorting Working Memory, Trail Making Test A/B. |
|  | Saenghong et al., 2012 | Delayed word recognition (% accuracy) | 2 months | Selected as the delayed recognition memory outcome entered in the episodic memory meta-analysis. | Other computerized cognitive battery outcomes and ERP components, including N100 and P300 amplitude/latency. |
|  | Bahrami et al., 2023 | Memory score from the Cognitive Abilities Task | After 3 menstrual cycles | Selected as the specific memory-domain score entered in the episodic memory meta-analysis. | Inhibitory control and selective attention, decision making, planning, sustained attention, social cognition, cognitive flexibility, and total cognitive ability. |
| Executive function/processing speed | Small et al., 2018 | Trail Making Test Part A | 18 months | Selected as the processing speed/attention-related measure entered in the executive function/processing speed meta-analysis. | Brief Visual Memory Test, Buschke Selective Reminding Test. |
|  | Rainey-Smith et al., 2016 | Wechsler Digit Symbol Scale | 12 months | Selected as the perceptual-motor speed/processing speed measure entered in the executive function/processing speed meta-analysis. | MoCA, RAVLT measures, COWAT, cognitive composite scores. |
|  | Santos-Parker et al., 2018 | NIH Toolbox Pattern Comparison Processing Speed Test | 12 weeks | Selected as the NIH Toolbox processing speed measure entered in the executive function/processing speed meta-analysis. | NIH Toolbox Flanker Inhibitory Control and Attention, Picture Sequence Memory, List Sorting Working Memory, Trail Making Test A/B. |
| Global cognition | Rainey-Smith et al., 2016 | Montreal Cognitive Assessment | 12 months | Selected as the global cognitive screening measure entered in the global cognition meta-analysis. | RAVLT measures, Wechsler Digit Symbol Scale, COWAT, cognitive composite scores. |
|  | Badakhshan et al., 2025 | Mini-Mental State Examination | 8 weeks | Selected as the global cognitive screening measure reported in the trial. | Clinical Dementia Rating. |
|  | Ringman et al., 2012 | Mini-Mental State Examination | 24 weeks | Selected as the global cognitive screening measure entered in the global cognition meta-analysis. | ADAS-Cog, NPI, ADCS-ADL, plasma and cerebrospinal fluid biomarkers. |
|  | Das et al., 2023 | Mini-Mental State Examination | 24 weeks | Selected as the global cognitive screening measure entered in the global cognition meta-analysis. | Geriatric Locomotive Function Scale, BDNF, Aβ42, tau, IL-6, and TNF-α. |
| Attention or inhibitory control | Santos-Parker et al., 2018 | NIH Toolbox Flanker Inhibitory Control and Attention Test | 12 weeks | Selected as the NIH Toolbox measure corresponding to inhibitory control and attention. | NIH Toolbox Pattern Comparison Processing Speed, Picture Sequence Memory, List Sorting Working Memory, Trail Making Test A/B. |
|  | Nakamura et al., 2025 | Complex Attention score from Cognitrax | 12 weeks | Selected because Complex Attention was the prespecified primary outcome of the trial. | Neurocognitive Index, Composite Memory, Verbal Memory, Visual Memory, Psychomotor Speed, Reaction Time, Cognitive Flexibility, Processing Speed, Executive Function, Working Memory, Sustained Attention, Simple Attention, Motor Speed, and critical flicker-fusion frequency. |
|  | Bahrami et al., 2023 | Inhibitory control and selective attention score from the Cognitive Abilities Task | After 3 menstrual cycles | Selected as the attention/inhibitory-control domain score entered in the attention/inhibitory control meta-analysis. | Memory, decision making, planning, sustained attention, social cognition, cognitive flexibility, and total cognitive ability. |

Abbreviations: ADAS-Cog, Alzheimer’s Disease Assessment Scale-Cognitive Subscale; ADCS-ADL, Alzheimer’s Disease Cooperative Study-Activities of Daily Living; Aβ42, amyloid-β42; BDNF, brain-derived neurotrophic factor; COWAT, Controlled Oral Word Association Test; ERP, event-related potential; IL-6, interleukin-6; MMSE, Mini-Mental State Examination; MoCA, Montreal Cognitive Assessment; NIH, National Institutes of Health; NIHTB, National Institutes of Health Toolbox; NPI, Neuropsychiatric Inventory; RAVLT, Rey Auditory Verbal Learning Test; TNF-α, tumour necrosis factor-alpha.
