# Supplementary material for: Effects of Zingiberaceae-derived interventions on memory-related and other cognitive outcomes in adults: a systematic review and meta-analysis
Source: Front Nutr. 2026 May 11;13:1834167. doi: 10.3389/fnut.2026.1834167 (PMC13198985; doi:10.3389/fnut.2026.1834167)
Supplement: Supplementary file 4 [file Table_4.docx]

Table S4. GRADE Summary of Findings for cognitive outcomes included in the meta-analysis.

| **Certainty assessment** | | | | | | | **№ of patients** | | **Effect** | | **Certainty** | **Importance** |
| --- | --- | --- | --- | --- | --- | --- | --- | --- | --- | --- | --- | --- |
| **№ of studies** | **Study design** | **Risk of bias** | **Inconsistency** | **Indirectness** | **Imprecision** | **Other considerations** | **Zingiberaceae-derived interventions** | **Placebo or control intervention** | **Relative (95% CI)** | **Absolute (95% CI)** |  |  |
| **Episodic memory (assessed with: Brief Visual Memory Test – Delay; RAVLT delayed recall; NIH Toolbox Picture Sequence Memory; delayed word recognition; Memory score from the Cognitive Abilities Task)** | | | | | | | | | | | | |
| 5 | randomised trials | serious^a^ | serious^b^ | not serious | serious^c^ | none | 157 | 175 | - | SMD **0.57 SD higher** (0.13 higher to 1.02 higher) | ⨁◯◯◯ Very low^a,b,c^ | CRITICAL |
| **Executive function and processing speed (assessed with: Wechsler Digit Symbol Scale; NIH Toolbox Pattern Comparison Processing Speed Test; Trail Making Test Part A)** | | | | | | | | | | | | |
| 3 | randomised trials | serious^d^ | serious^e^ | not serious | serious^f^ | none | 80 | 95 | - | SMD **0.02 SD lower** (0.71 lower to 0.67 higher) | ⨁◯◯◯ Very low^d,e,f^ | CRITICAL |
| **Global cognition (assessed with: Mini-Mental State Examination; Montreal Cognitive Assessment)** | | | | | | | | | | | | |
| 4 | randomised trials | serious^g^ | not serious | not serious | serious^h^ | none | 95 | 114 | - | SMD **0.06 SD higher** (0.21 lower to 0.33 higher) | ⨁⨁◯◯ Low^g,h^ | CRITICAL |
| **Attention or inhibitory control (assessed with: NIH Toolbox Flanker Inhibitory Control and Attention Test; Cognitrax Complex Attention; inhibitory control and selective attention score from the Cognitive Abilities Task)** | | | | | | | | | | | | |
| 3 | randomised trials | serious^i^ | not serious | serious^j^ | serious^k^ | none | 117 | 119 | - | SMD **0.07 SD lower** (0.33 lower to 0.18 higher) | ⨁◯◯◯ Very low^i,j,k^ | CRITICAL |

#### CI: confidence interval; MMSE: Mini-Mental State Examination; MoCA: Montreal Cognitive Assessment; NIH: National Institutes of Health; RAVLT: Rey Auditory Verbal Learning Test; SD: standard deviation; SMD: standardised mean difference.

#### Explanations

a. Downgraded one level because some studies contributing to this outcome had methodological concerns, including predictable allocation in Bahrami et al., insufficiently described allocation concealment in Saenghong et al., and missing outcome data concerns in Rainey-Smith et al.

b. Downgraded one level because substantial heterogeneity was observed (I² = 73.02%), with variability in the magnitude of effects across studies.

c. Downgraded one level because the evidence was based on a small number of studies and participants, and the confidence interval was relatively wide.

d. Downgraded one level because contributing studies raised methodological concerns, particularly related to missing outcome data, selection of the reported result, and incomplete reporting of allocation concealment.

e. Downgraded one level because substantial heterogeneity was observed (I² = 79.10%), with effects varying in direction and magnitude across studies.

f. Downgraded one level because the estimate was based on only three studies and the confidence interval was wide and compatible with benefit, no effect, or harm.

g. Downgraded one level because some studies contributing to this outcome had high risk of bias or concerns related to missing outcome data and completer-based analyses, particularly Rainey-Smith et al. and Ringman et al.

h. Downgraded one level because the evidence was based on a small number of studies and participants, and the confidence interval crossed the line of no effect.

i. Downgraded one level because some contributing studies had methodological concerns, including predictable allocation in Bahrami et al. and selection of the reported result or incomplete reporting in other studies.

j. Downgraded one level because the pooled domain combined related but not identical constructs, including inhibitory control, selective attention, and Complex Attention from Cognitrax.

k. Downgraded one level because the estimate was based on only three studies and the confidence interval crossed the line of no effect.
