# Supplementary material for: Effects of Zingiberaceae-derived interventions on memory-related and other cognitive outcomes in adults: a systematic review and meta-analysis
Source: Front Nutr. 2026 May 11;13:1834167. doi: 10.3389/fnut.2026.1834167 (PMC13198985; doi:10.3389/fnut.2026.1834167)
Supplement: Supplementary Figure 1 — Cochrane Risk of Bias 2 (RoB 2) assessment for parallel-group randomized controlled trials assessed under intention-to-treat (ITT) assumptions. [file Image_1.pdf]

Study	D1	D2	D3	D4	D5	Overall
Rainey-Smith et al., 2016	!	+	-	+	+	-
Santos-Parker et al., 2018	!	+	!	+	+	!
Badakhshan et al., 2025	!	+	+	+	+	!
Gimblet et al., 2024	+	+	!	+	+	!
Laksmidewi et al., 2024	!	+	-	+	!	-
Lee et al., 2014	!	!	!	+	!	!
Saenghong et al., 2012	!	+	!	+	!	!

+ Low risk

! Some concerns

- High risk

D1 Randomisation process

D2 Deviations from the intended interventions

D3 Missing outcome data

D4 Measurement of the outcome

D5 Selection of the reported result
