# Supplementary material for: Effects of Zingiberaceae-derived interventions on memory-related and other cognitive outcomes in adults: a systematic review and meta-analysis
Source: Front Nutr. 2026 May 11;13:1834167. doi: 10.3389/fnut.2026.1834167 (PMC13198985; doi:10.3389/fnut.2026.1834167)
Supplement: Supplementary Figure 2 — Cochrane Risk of Bias 2 (RoB 2) assessment for parallel-group randomized controlled trials assessed under per-protocol (PP) assumptions. [file Image_2.pdf]

Study	D1	D2	D3	D4	D5	Overall
Ringman et al., 2012	+	+	-	+	+	-
Cox et al., 2015	+	+	+	!	!	!
Small et al., 2018	+	+	+	+	!	!
Kuszewski et al., 2020	+	+	!	+	!	!
Cox et al., 2020	+	+	+	+	+	+
Khanna et al., 2022	+	+	+	+	+	+
Bahrami et al., 2023	-	+	!	+	!	-
Nakamura et al., 2025	!	+	!	+	+	!
Das et al., 2023	!	+	+	+	!	!
Alimadadi et al., 2022	!	+	+	+	!	!

+ Low risk

! Some concerns

- High risk

D1	Randomisation process
D2	Deviations from the intended interventions
D3	Missing outcome data
D4	Measurement of the outcome
D5	Selection of the reported result
