# Supplementary material for: Effects of Zingiberaceae-derived interventions on memory-related and other cognitive outcomes in adults: a systematic review and meta-analysis
Source: Front Nutr. 2026 May 11;13:1834167. doi: 10.3389/fnut.2026.1834167 (PMC13198985; doi:10.3389/fnut.2026.1834167)
Supplement: Supplementary Figure 3 — Cochrane Risk of Bias 2 (RoB 2) assessment for the crossover randomized controlled trial. [file Image_3.pdf]

| **Article** | **D1** | **DS** | **D2** | **D3** | **D4** | **D5** | **Overall** |
| --- | --- | --- | --- | --- | --- | --- | --- |
| Srivastava et al., 2017 | + | + | + | + | + | ! | ! |

| | |
| --- | --- |
| + | Low risk |
| ! | Some concerns |
| - | High risk |

| | |
| --- | --- |
| D1 | Randomisation process |
| DS | Bias arising from period and carryover effects |
| D2 | Deviations from the intended interventions |
| D3 | Missing outcome data |
| D4 | Measurement of the outcome |
| D5 | Selection of the reported result |
