# Supplementary figures and images for: Effects of Zingiberaceae-derived interventions on memory-related and other cognitive outcomes in adults: a systematic review and meta-analysis
Source: Front Nutr. 2026 May 11;13:1834167. doi: 10.3389/fnut.2026.1834167 (PMC13198985; doi:10.3389/fnut.2026.1834167)

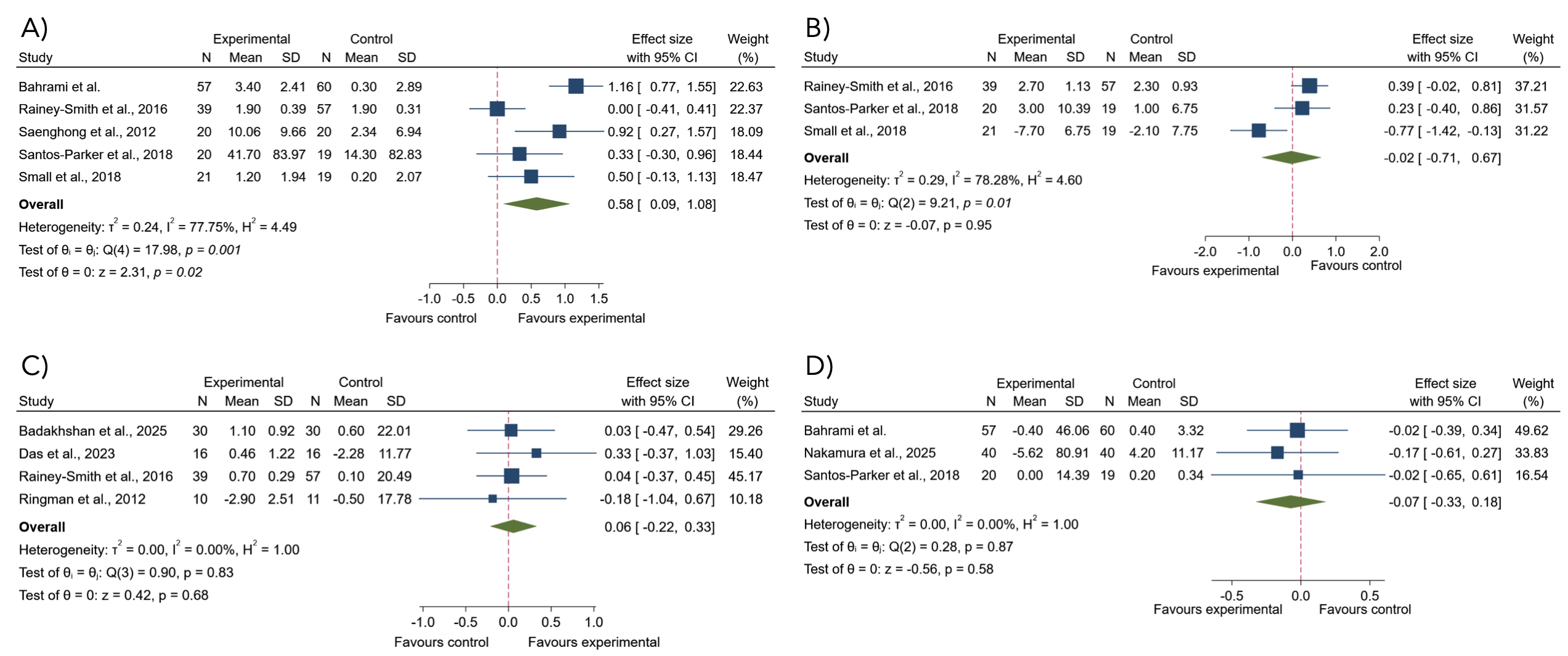

Supplement: Supplementary Figure 4 — Sensitivity analysis forest plots for the cognitive outcomes included in the meta-analysis. (A) Episodic memory; (B) executive function/processing speed; (C) global cognition; (D) attention or inhibitory control. [file Image_4.png]

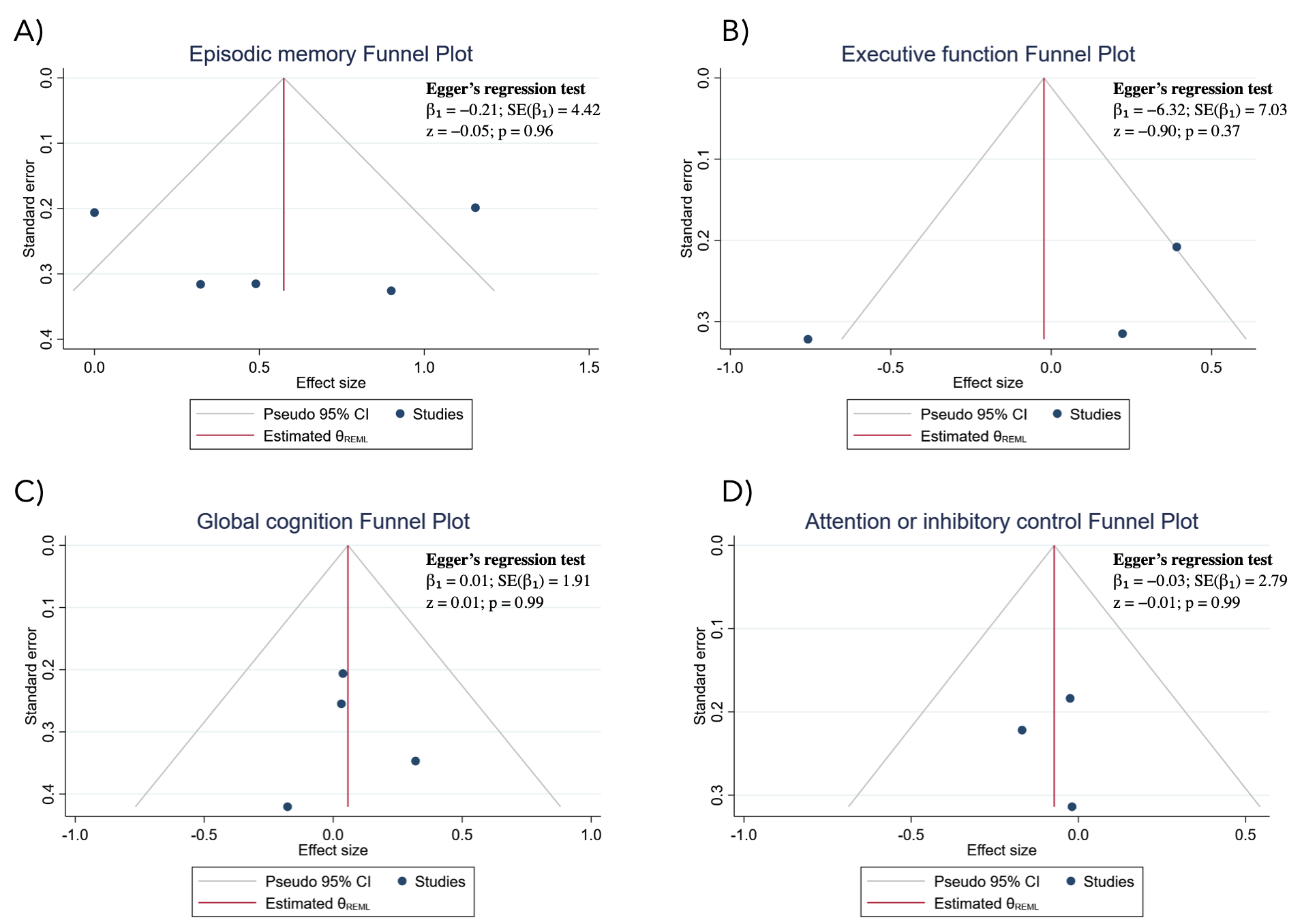

Supplement: Supplementary Figure 5 — Funnel plots with Egger’s regression tests for the same cognitive outcomes. (A) Episodic memory; (B) executive function; (C) global cognition; (D) attention or inhibitory control. [file Image_5.png]
